# An efficient method for tissue-specific protoplast isolation suitable for single-cell RNA sequencing and transient gene expression analysis in saffron (*Crocus sativus* L.)

**DOI:** 10.1186/s12896-026-01187-1

**Published:** 2026-07-08

**Authors:** Xiaoyuan Xi, Jia Song, Mengqing Feng, Xiaodong Qian, Heng Sun, Liqin Li

**Affiliations:** 1 TCM (Traditional Chinese Medicine) Key Laboratory Cultivation Base of Zhejiang Province for the Development and Clinical Transformation of Immunomodulatory drugs, Huzhou Central Hospital, Fifth School of Clinical Medicine of Zhejiang, Chinese Medical University, 1558 Sanhuan North Road, Huzhou, Zhejiang 313000 China; 2 Affiliated Huzhou Hospital, Zhejiang University School of Medicine, Huzhou, Zhejiang China; 3 Huzhou Central Hospital, Affiliated Central Hospital of Huzhou University, Huzhou, Zhejiang China; 4 School of Pharmacy, Hangzhou Normal University, Hangzhou, Zhejiang China

**Keywords:** Saffron, Tissue-specific protoplast isolation, Efficient, Single-cell RNA sequencing, Transient gene expression

## Abstract

**Background:**

Protoplasts have been widely utilized in tissue culture, single-cell RNA sequencing, and transient gene expression analyses. However, the primary challenge in their preparation lies in the efficient removal of rigid cell walls to obtain high-quality plant cells. The aim of this study is to establish an effective method for protoplast isolation from saffron (*Crocus sativus* L.), thereby accelerating advances in molecular biology research in this species.

**Result:**

Tissue-specific protoplasts were successfully isolated from apical buds, petals, leaves, and roots of saffron, with yields of approximately 8.81 × 10^6^, 1.36 × 10⁷, 7.84 × 10⁵, and 5.69 × 10⁵/g FW, and viabilities of ~ 95%, ~ 94%, ~ 96%, and ~ 61%, respectively. The optimal enzymatic digestion conditions varied by tissue type: apical buds (0.6 M D-mannitol, 1.5% cellulase, 0.5% macerozyme, 28 °C, 3 h); roots (0.7 M D-mannitol, 2.0% cellulase, 0.5% macerozyme, 0.5% pectinase, 28 °C, 4 h); petals (0.7 M D-mannitol, 1.5% cellulase, 0.5% macerozyme, 28 °C, 3.5 h); and mesophyll (0.7 M D-mannitol, 1.5% cellulase, 0.5% macerozyme, 28 °C, 4 h). The quality of the protoplasts was affected by developmental stages and sampling locations. Furthermore, two high-quality single-cell libraries were constructed using protoplasts from non-flowering and flowering apical buds, respectively. We successfully visualized the subcellular localization of GFP in the mesophyll protoplasts.

**Conclusion:**

This study presents the first detailed report on protoplast isolation, evaluation, and application in saffron. By overcoming the technical challenges of tissue-specific protoplast isolation from diverse organs, we established a highly efficient method that enables both the development of novel germplasm and mechanistic insights into saffron organogenesis, growth, and development.

**Graphical Abstract:**

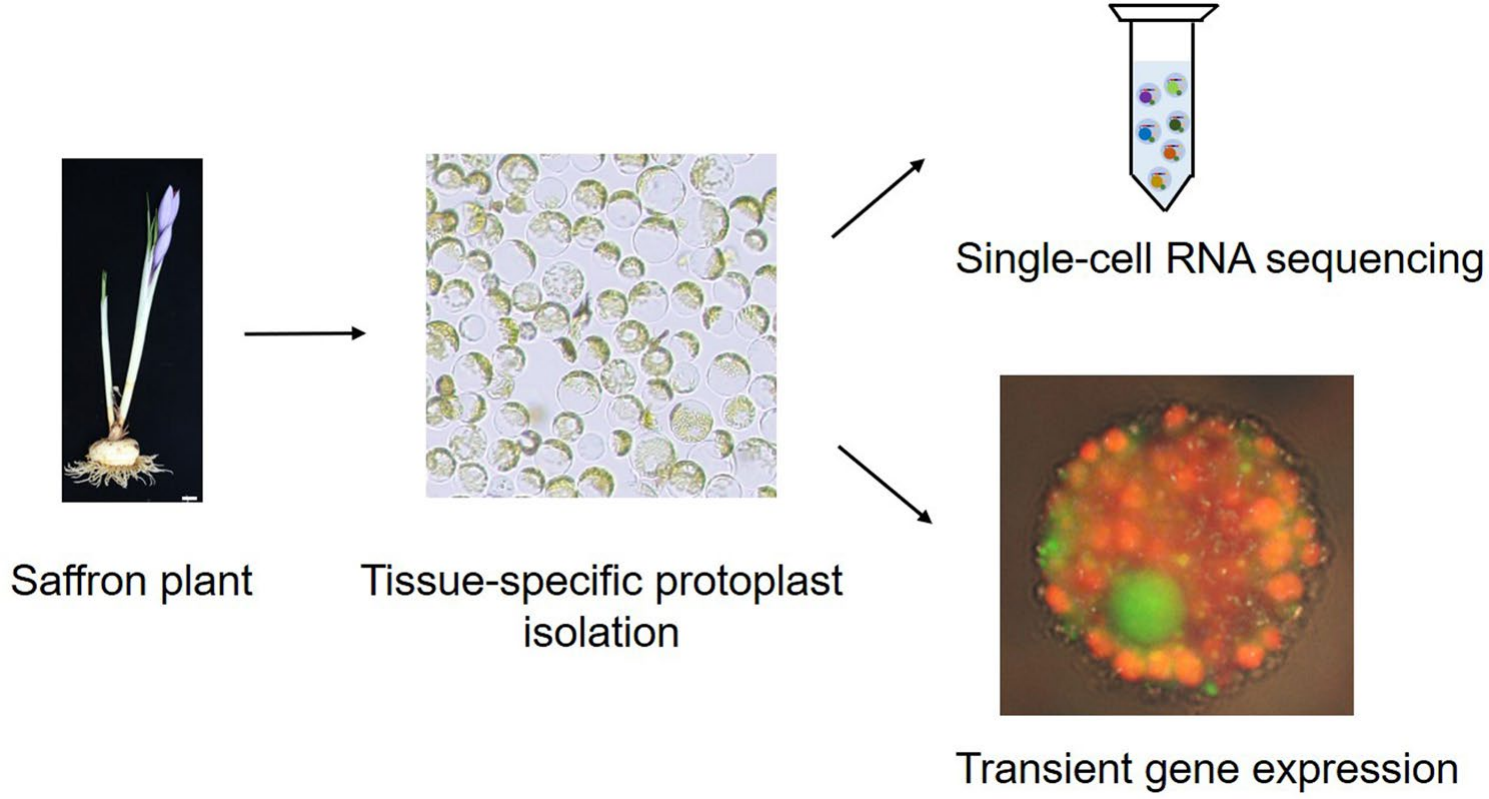

**Supplementary Information:**

The online version contains supplementary material available at 10.1186/s12896-026-01187-1.

## Background

Protoplasts, plant cells without cell walls, play a crucial role in plant biology [1, 2]. Their totipotency, rapid growth, and synchrony have attracted the interest of botanists [2, 3]. Protoplast-based single-cell sequencing reveals developmental trajectories and cellular diversity, facilitating the identification of cell types and fate determination [4–6]. The protoplast transient expression system allows for rapid studies on protein subcellular localization, protein-protein and protein-DNA interactions, and genome editing [7–14], thereby accelerating advances in molecular biology [15–17]. Furthermore, sexual barriers can be overcome through protoplast fusion or somatic cell hybridization [18–20].

The yield of saffron (*Crocus sativus* L.) is intricately linked to the nutritional status of corms [21]. Therefore, it is of great significance to elucidate the mechanism of how the roots or leaves convert water, inorganic salts and light signals into nutrients that support corm growth and development. The petals of saffron possess both medicinal and ornamental value [22–24]. It holds considerable application prospects for identifying the key genes that influence the medicinal efficacy or petal color, and generating novel germplasm with excellent traits. In the process of the floral organ development, the more floral primordia formed, the more flowers will be produced [25–27]. A crucial question in developmental biology is understanding how the information on DNA sequences is translated into physiological and morphological traits for determining floral primordia formation. Excitingly, this question can be answered by protoplast-based technology, such as single-cell RNA sequencing (scRNA-seq). scRNA-seq can help us understand the molecular mechanisms governing the stepwise cell fate specification and regulating organ organization or patterning, and stress response during plant and animal development [28–33]. To the present, scRNA-seq in plants has not been used as widely as in animals, particularly because the presence of cell walls inhibits the dissociation of intact and viable individual cells and impedes mature mRNA release [5]. However, the plant cell wall differs in thickness and composition depending on plant species, tissues, developmental stages, and environmental conditions [29]. Hence, it is essential to develop tissue-specific protoplast isolation methods to successfully generate high-yield and high-quality protoplasts in saffron.

Here, we describe an efficient method for tissue-specific protoplast isolation in saffron. Apical buds at the flowering transition stage, light purple petals, the roots of 0.5–2 cm from the root tip and the second segment of leaves with a length of 10–15 cm were the best stages and sampling locations to prepare apical bud, petal, root and mesophyll protoplasts, respectively. The protoplast yield was influenced by the enzymatic compositions and proportions, digestion time, and D-mannitol concentration. We constructed two high-quality single-cell libraries using apical bud protoplasts and visualized the subcellular localization of GFP in the mesophyll protoplasts. Our work aims to establish an efficient method for isolating tissue-specific protoplasts from different organs of saffron (*Crocus sativus* L.). This method not only facilitates the creation of novel germplasm lines with advantageous traits but also contributes to basic research in molecular biology aimed at shedding light on the complex regulatory mechanisms in saffron.

## Methods

### Plant materials and growth conditions

Saffron plants (Fig. S1A) were cultivated in the experimental fields in the southern suburbs of Huzhou City, Zhejiang Province, China. The cultivation conditions were as described previously [34]. The life cycle of saffron can be divided into two stages: the reproductive growth occurs from June to November, followed by a vegetative growth from December to May of the next year, and the daughter corms are generated at the base of shoots. In April or May, the leaves senesce and wither, and the corms go into dormancy. Apical buds at the flowering transition stage, the roots of 0.5–2 cm from the tip, light purple petals and the second segment of leaves with a length of 10–15 cm were used to isolate protoplasts.

### Protoplast isolation and purification

The protoplasts were isolated following the protocols reported in previous studies [9] with some modifications. Tissue materials, including apical buds, root tips, petals and leaves were used for tissue-specific protoplast isolation (Fig. S1C, S2C, S3, S4). The samples were surface sterilized by dipping into 75% (v/v) alcohol for 30 s, followed by three washes in sterilized water. Approximately 0.5 g fresh weight of apical buds, roots, petals and leaves were cut into 1–2 mm thin strips (Fig. S1B, S2B, S3A, S4E). 10 mL enzymatic solution was added to the petri dish, vacuumed for 30 min at -0.1 MPa, and then incubated at 28 °C in the dark for enzymolysis. The enzymatic solution contained 20 mM 2-(N-Morpholino) ethanesulfonic acid (pH 5.5–5.8) with different concentrations of D-mannitol (0.4, 0.5, 0.6 and 0.7 M), cellulase R -10 (Yakult, Japan) (0.5%, 1.0%, 1.5% and 2.0% (w/v)), macerozyme R -10 (Yakult, Japan) (0.5% and 1.0% (w/v)) and 20 mM KCl. The solution was warmed at 55℃ for 10 min and cooled to room temperature. Then, 10 mM CaCl_2_ (Sigma) and 0.1% (w/v) bovine serum albumin (Sigma) were added. All enzymatic solutions were filter-sterilized through a 0.45 μm syringe filter (Millipore Sigma) and stored at 4 °C for later use. When isolating protoplasts from apical buds and roots, it was necessary to pre-treat the strips of apical buds and roots with pretreatment buffer for 30 min. The components of the pretreatment buffer were the same as those of the enzymatic solution, except that the enzymes were not added. In addition to cellulase and macerozyme, 0.5% and 1.0% (w/v) pectinase were also required in the enzymatic solution to isolate protoplasts from roots. After incubation at 28 °C in the dark for different durations (2, 2.5, 3, 3.5, 4, and 5 h), the released protoplasts were harvested.

Mesophyll and petal protoplasts were purified by filtration through a cell sieve, centrifugation and natural sedimentation. After digestion, the enzyme mixture containing protoplasts was diluted with an equal volume of wash solution (W5) that contained 154 mM NaCl (Sigma), 125 mM CaCl_2_, 5 mM KCl, and 2 mM MES (pH 5.8). The protoplast-containing solution was filtered through 100 μm, 70 μm and 40 μm cell sieves into a 50 mL round-bottomed centrifuge tube and centrifuged at 150×g for 2 min to pellet the protoplasts. Following this, the supernatant was removed as much as possible and the protoplast pellet was resuspended in an appropriate amount of W5 solution. Subsequently, the yield, viability and integrity of protoplasts were estimated. Apical bud and root protoplasts were purified by filtration through a cell sieve and sucrose gradient centrifugation. After the protoplast-containing solution was filtered with 70 μm and 40 μm cell sieves and centrifuged at 150×g for 5 min, resuspending the protoplast pellet carefully with 2 mL MMG solution that contained 4 mM MES (pH 5.8), 0.6 M D-mannitol (apical bud) or 0.7 M D-mannitol (root), 15 mM MgCl_2_. The protoplast-containing MMG solution was transferred to 6 mL 52% sucrose solution, and centrifuged at 150×g for 5 min at room temperature. The step was repeated if more impurities were present. Nearly 2 mL purified protoplast suspension was at the sucrose-MMG interface. Then, the yield, viability and integrity of protoplasts were estimated.

### Protoplast yield, cell viability and integrity evaluation

The yield was evaluated as the number of protoplasts released per gram of fresh (protoplasts/g FW) of the sample. The protoplasts were counted under a microscope (OLYMPUS, IX73P1F, TOKYO 163–0914, JAPAN) using a haemocytometer. The viability of protoplasts was assessed by staining with 0.01% (w/v) fluorescein diacetate (FDA). The specific details were followed according to the instructions. Viable protoplasts with green fluorescence were visualized and photographed using a fluorescence microscope (Leica Microsystems CMS GmbH, Wetzlar, Germany, DM3000). The green fluorescence was detected with excitation at 488 nm and emission at 507 nm. The cell integrity was analyzed using trypan blue staining and photographed using the same microscope as cell counting. The details of trypan blue staining were carried out as described in the instructions [35]. The protoplast yield, cell viability and integrity were evaluated by examining at least three fields for each sample.

### Heat shock combined with PEG-mediated transformation of protoplasts

Heat shock combined with PEG-mediated protoplast transformation was conducted in saffron, referring to a modified PEG-mediated protocol [9]. For each transformation, 15–20 µg plasmid (about 10 µL) was added to 100 µL mesophyll protoplast suspension (resuspended in prechilled MMG solution) with a density of 1 × 10^5^ − 1 × 10^6^/mL. An equal volume (110 µL) of freshly prepared PEG-CaCl_2_ solution composed of 100 mM CaCl_2_, 0.4 M D-mannitol, and 40% PEG4000 was added and mixed well. The mixture was incubated at 42℃ for 10 min. The transformation was stopped by adding 440 µL W5 solution, followed by centrifugation at 150×g for 2 min, after which the supernatant was removed. The transfected protoplasts were resuspended with 1 mL WI solution (4 mM MES, 0.7 M D-mannitol, 20 mM KCl, pH 5.8) and incubated at 18 °C in the dark for 12–24 h.

### Construction of scRNA-seq library

Cell suspensions were loaded into the 10×Genomics Chromium single-cell microfluidics device to generate single-cell gel beads-in-emulsion (GEMs). The scRNA-seq library was generated with the Single Cell 3’ Library & Gel Bead Kit V3 (10×Genomics, PN-1000075) according to the manufacturer’s instructions. The barcoded, reverse-transcribed full-length cDNAs were amplified by PCR, and the resulting products were ligated to sequencing adapters. The paired-end library was constructed and sequenced using the Illumina NovaSeq 6000 platform. Qualitative analysis of the DNA library was performed by an Agilent 4150 Bioanalyzer, while its concentration was measured by Qubit.

### Statistical analysis

All experiments were performed in triplicate. Statistical analysis was conducted using one-way analysis of variance (ANOVA) with GraphPad Prism 8 software. Data are presented as mean ± standard error of the mean (SEM) from three independent experiments. Differences between treatments were considered significant at *P < 0.05, **P < 0.01 and ***P < 0.001, respectively.

## Results

### Optimized an efficient method for apical bud protoplast isolation in *Crocus sativus* L.

**Fig. 1:**
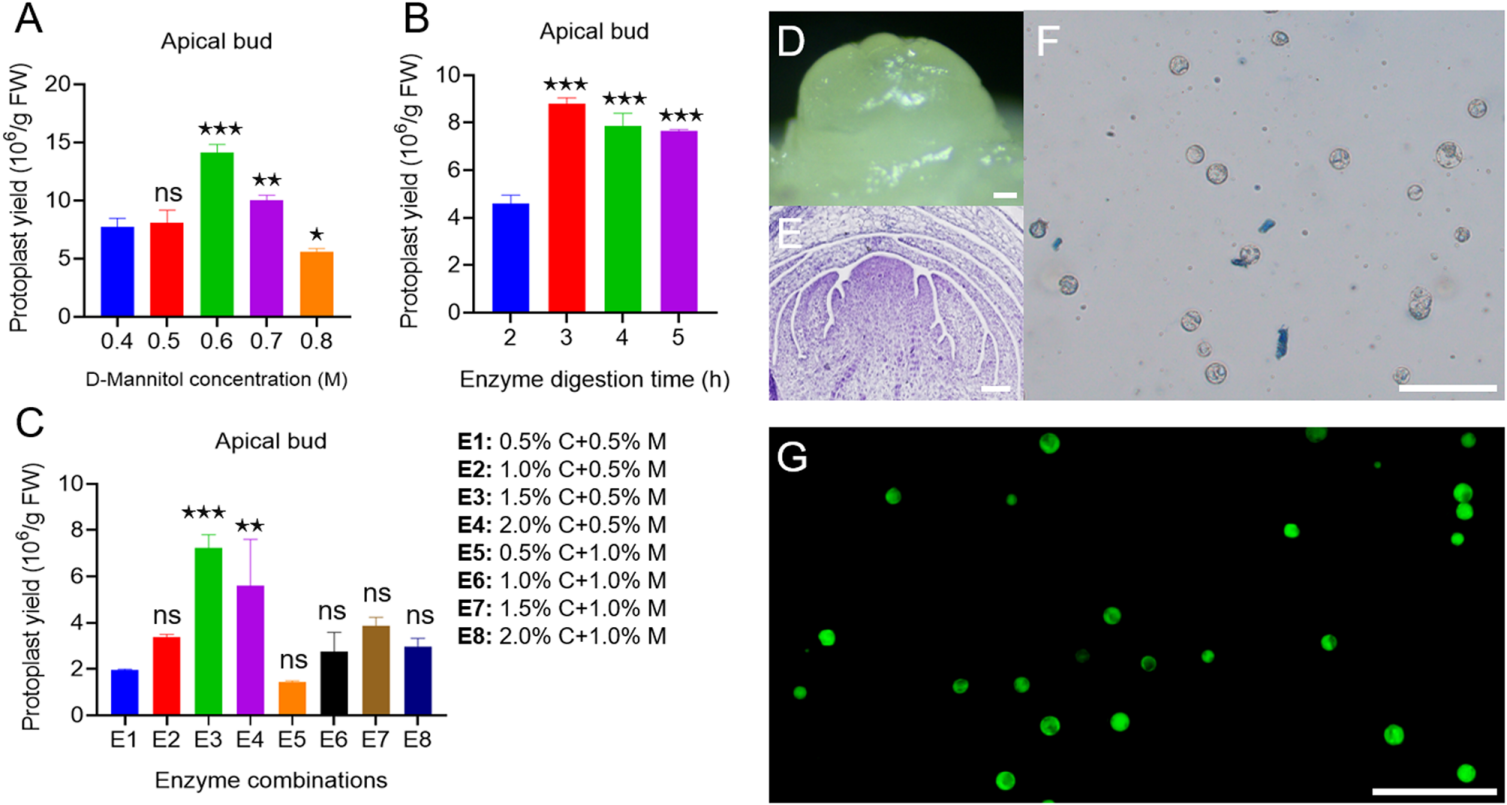
Protoplast isolation from apical buds of saffron. Factors including (**A**) D-mannitol concentrations, (**B**) digestion durations, and (**C**) enzyme combinations were investigated to optimize the yield. C: cellulase R-10, M: macerozyme R-10. Data are means ± SD of three biological replicates. *P < 0.05, **P < 0.01, ***P < 0.001; ns, not significant. (**D**) Morphological observations of apical bud at the flowering transition stage were photographed using a stereoscope (SOPTOP SZN). (**E**) Paraffin sections of apical buds at the flowering transition stage for morphological observation. (**F**) The cell membrane integrity was analyzed using trypan blue staining. Cells that were not stained blue indicated good membrane integrity. (**G**) The cell viability was assessed by FDA staining. The protoplasts with the green fluorescence signals were energetic cells and were imaged under the GFP channel. Scale bars = 0.5 mm for (**D**) and (**E**), and scale bars = 100 μm for (**F**) and (**G**)

To isolate protoplasts from apical bud at the flowering transition stage (Fig. 1D and E), we conducted a systematic examination of various factors on the yield, including D-mannitol concentration (Fig. 1A), enzyme combinations (Fig. 1C) and digestion duration (Fig. 1B). Due to the high starch content surrounding the apical bud tissues, it was necessary to wash the excised apical buds cut from corms 3–5 times with sterile water to ensure the efficiency of protoplast dissociation. The protocols for protoplast isolation vary with the species, tissue types and developmental stages. In this process, different pretreatment methods were conducted to optimize the yield and quality of the isolated protoplasts [1]. In our study, a pretreatment buffer containing the same components as the enzymatic solution but lacking the enzymes was applied to the apical buds for 30 min at room temperature to harvest more viable protoplasts. Subsequently, gradient concentrations of D-mannitol, different enzyme combinations and digestion durations were optimized to screen for the best conditions of releasing protoplasts. The results suggested that increasing D-mannitol concentrations led to increased protoplast yields, whereas excessive concentrations reduced yields, with the maximum yield achieved at 0.6 M (Fig. 1A). Significant differences were observed in protoplast yields depending on the enzyme combinations and digestion time. The highest protoplast yield was 8.81 × 10^6^/g FW by employing a combination of 1.5% cellulase and 0.5% macerozyme for a duration of 3 h (Fig. 1B and C). FDA and trypan blue staining were performed to assess the cell viability and integrity (Fig. 1F and G). Protoplasts were considered viable if they exhibited green fluorescence or were not stained blue. The results showed that 95% of cells were healthy and energetic, with intact cell membranes. In conclusion, the optimal conditions for releasing apical bud protoplasts were using an enzymatic solution containing 0.6 M D-mannitol, 1.5% cellulase and 0.5% macerozyme, with digestion for 3 h (Fig. 1A, B and C).

### Optimized an efficient method for root protoplast isolation in *Crocus sativus* L.

**Fig. 2:**
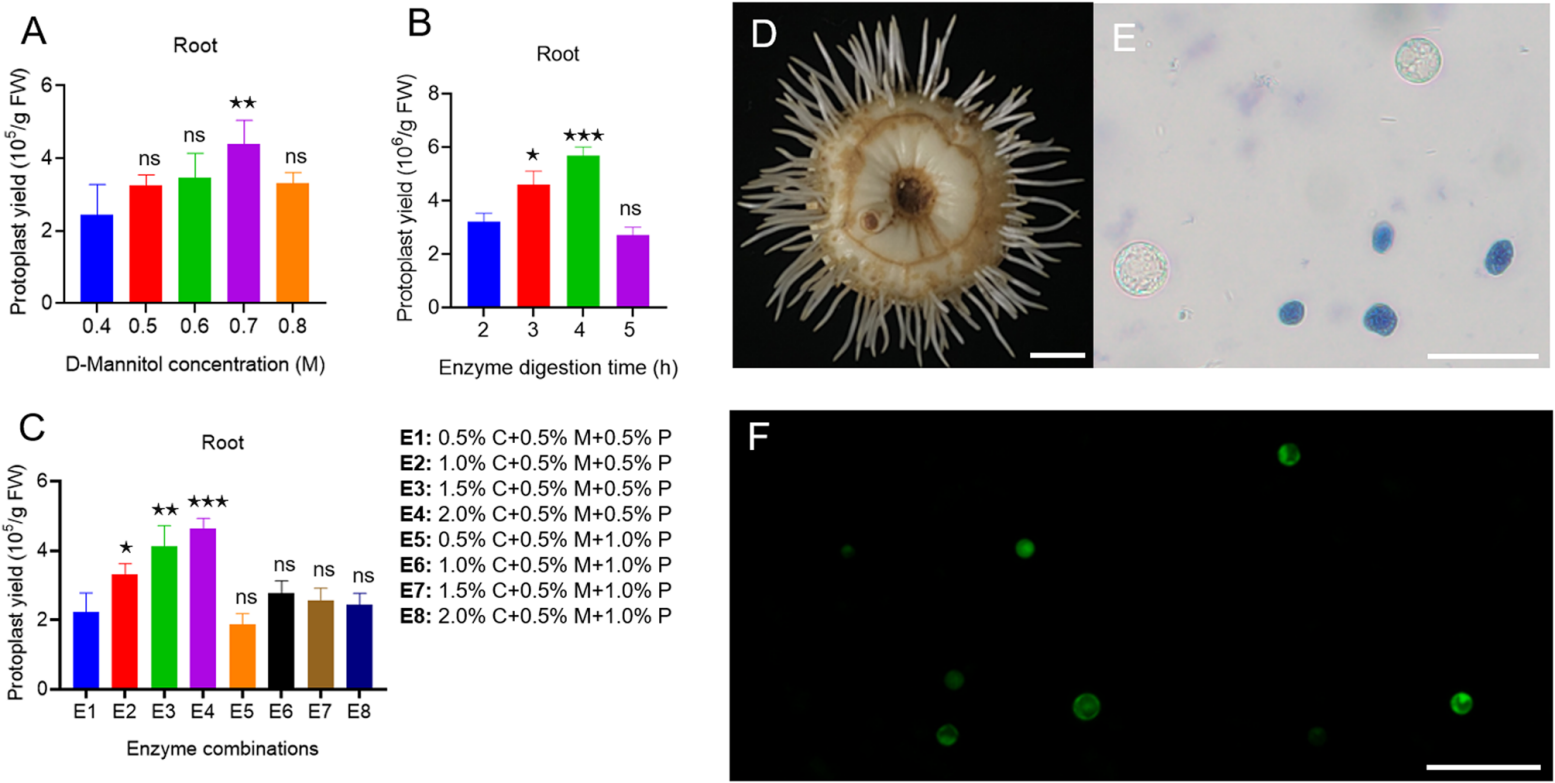
Protoplast isolation from fibrous roots of saffron. Different (**A**) D-mannitol concentrations and (**C**) enzyme combinations in the enzymatic solutions digested root tip tissues for (**B**) different durations to optimize the yield. C: cellulase R-10, M: macerozyme R-10. P: pectinase. Data are means ± SD of three biological replicates. *P < 0.05, **P < 0.01, ***P < 0.001; ns, not significant. (**D**) 1-week-old fibrous roots of saffron plants were photographed by a digital single lens reflex camera (DSLR, Canon, DS126321). Scale bar = 1 cm. (**E**) The cell membrane integrity was tested using trypan blue staining. Protoplasts from the root tended to produce a high proportion of broken and shrunken cells. (**F**) The cell viability assay was performed by FDA staining. The protoplasts with the green fluorescence signals had better viability. Scale bars = 100 µm for (**E**) and (**F**)

Fibrous roots of 0.5–1.5 cm from the root tip were collected for root protoplast isolation (Fig. 2D). The pretreatment method was consistent with that used for apical bud protoplasts. No matter what combinations of cellulase and macerozyme were used, the yield of protoplasts did not fluctuate greatly (Fig. S2A). Consequently, we added pectinase to the enzymatic solution. Eight combinations of three enzymes were used to calculate the yield of protoplasts. The yield of protoplasts increased and then decreased across eight treatments of different enzyme combinations, and the highest yield was 4.62 × 10^5^/g FW (Fig. 2C). Additionally, the effects of D-mannitol concentration and digestion time on protoplast yield were also optimized. Similar trends were observed, and the highest yield was obtained when samples were treated with 0.7 M D-mannitol and digested for 4 h (Fig. 2A and B). This combination of 2.0% cellulase, 0.5% macerozyme and 0.5% pectinase digested for 4 h gave a total yield of 5.69 × 10^5^/g FW. The cell viability was detected by FDA and trypan blue staining (Fig. 2). Protoplasts exhibiting green fluorescence or no blue staining were healthy and energetic cells (Fig. 2E and F). Compared to protoplasts from other tissues, the yield of protoplasts with good cell viability isolated from roots was lower, accounting for approximately 61%. In conclusion, optimal root protoplast isolation was achieved using an enzymatic solution of 0.7 M D-mannitol, 2.0% cellulase, 0.5% macerozyme, and 0.5% pectinase, with 4 h digestion (Fig. 2A, B and C).

### Optimized an efficient method for petal protoplast isolation in *Crocus sativus* L.

**Fig. 3:**
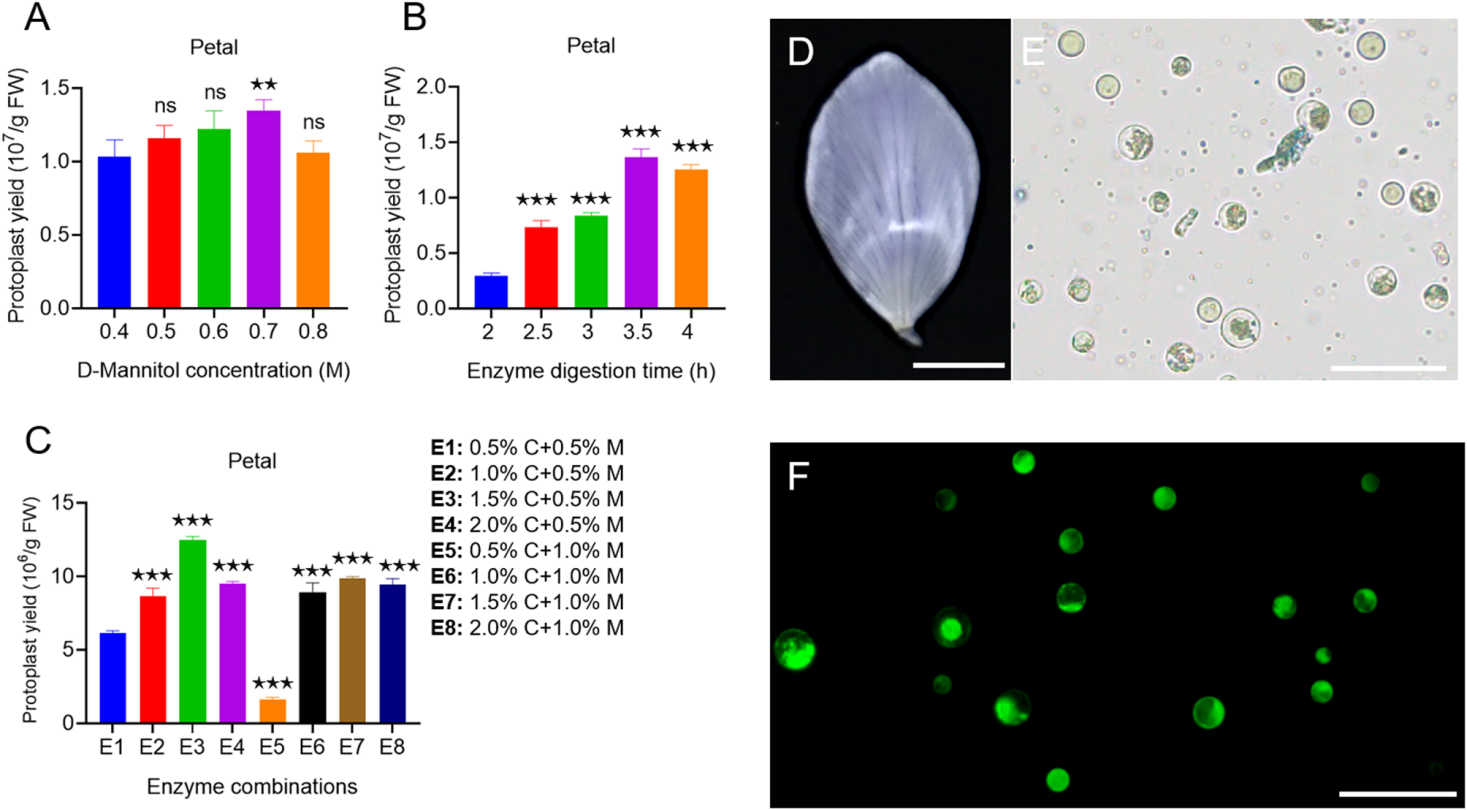
Protoplast isolation from petals of saffron. Effects of (**A**) D-mannitol concentration, (**B**) digestion duration, and (**C**) enzyme combinations on protoplast yield from petals. C: cellulase R-10, M: macerozyme R-10. Data are means ± SD of three biological replicates. *P < 0.05, **P < 0.01, ***P < 0.001; ns, not significant. (**D**) Light purple petals used for petal protoplast isolation were photographed by a digital single lens reflex camera (DSLR, Canon, DS126321). Scale bar = 0.5 cm. (**E**) Trypan blue staining was performed to test the cell membrane integrity, and the protoplasts that were not stained blue possessed an intact cell membrane. (**F**) The cell viability was measured by FDA staining. The protoplasts with the green fluorescence signals were healthy cells. Scale bars = 100 μm for (**E**) and (**F**)

White petals have a high moisture content, which will lead to protoplasts rupture easily (Fig. S3C and D). Protoplasts from dark purple petals have a high pigment content (Fig. S3E and F), especially anthocyanin, which will interfere with molecular biology experiments such as protein subcellular localization and single-cell sequencing analysis. Light purple petals are the best stage for isolating protoplasts (Fig. S3A and B). Various factors, such as different D-mannitol concentrations, enzyme combinations and digestion durations, were optimized to harvest high-yield and high-quality protoplasts (Fig. 3A, B and C). As the concentration of D-mannitol increased, the protoplast yield initially increased and then decreased. Specifically, when the D-mannitol concentration reached 0.7 M, there was a significant increase in the protoplast yield, reaching 1.34 × 10^7^/g FW (Fig. 3A). Eight combinations of cellulase and macerozyme with different ratios were tested to determine the optimal enzymatic composition. Significant differences were observed among different combinations, and when E3 combination was used, the protoplasts yield reached 1.25 × 10^7^/g FW (Fig. 3C). The highest yield of 1.36 × 10^7^/g FW was obtained when light purple petals were digested with an enzymatic solution containing 0.7 M D-mannitol and E3 enzyme combination for 3.5 h (Fig. 3A, B and C). The cell viability and integrity were determined by the FDA and trypan blue staining. The results showed that 94% of the cells were healthy and energetic (Fig. 3E and F). In general, the optimal conditions for isolating petal protoplasts involved an enzymatic solution containing 0.7 M D-mannitol, 1.5% cellulase and 0.5% macerozyme, with a digestion time of 3.5 h (Fig. 3A, B and C).

### Optimized an efficient method for mesophyll protoplast isolation in *Crocus sativus* L.

**Fig. 4:**
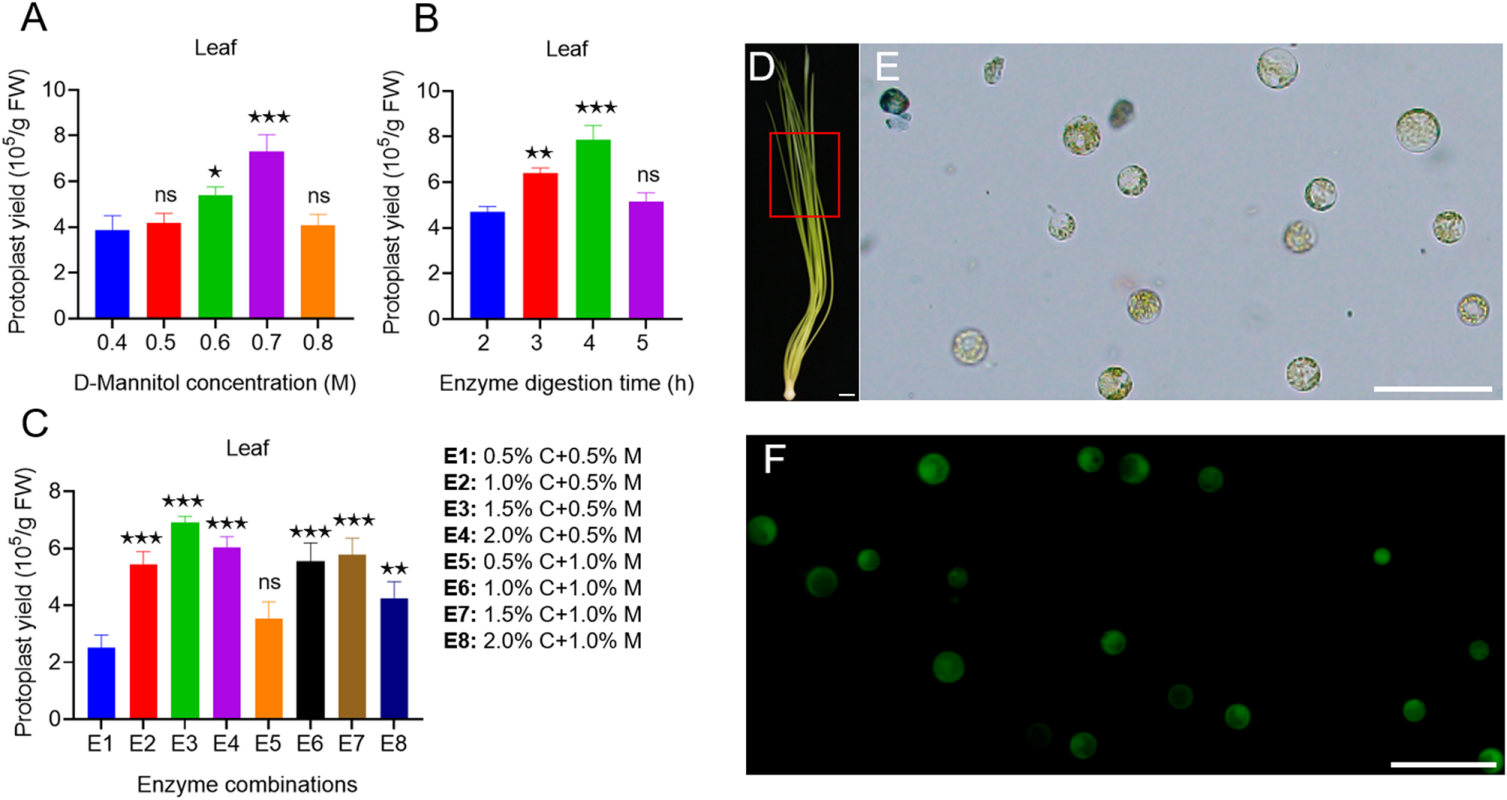
Isolation of saffron mesophyll protoplast. A method was established to optimize the yield of mesophyll protoplasts. This method used enzymatic solutions with different (**A**) D-mannitol concentrations and (**C**) enzyme combinations to digest the second segment of leaves for (**B**) varying durations. C: cellulase R-10, M: macerozyme R-10. Data are means ± SD of three biological replicates. *P < 0.05, **P < 0.01, ***P < 0.001; ns, not significant. (**D**) The mature leaves of saffron were photographed by a digital single lens reflex camera (DSLR, Canon, DS126321). The red box represents the best segment for isolating mesophyll protoplasts. Scale bar = 1 cm. (**E**) Trypan blue staining and (**F**) FDA staining assays were carried out to determine the protoplasts’ integrity and viability. The protoplasts that were not stained blue or had green fluorescence signals had better viability. Scale bars = 100 μm for (**E**) and (**F**)

Usually, the length of mature saffron leaves is up to 40–70 cm [30], but only a small segment of the leaf is suitable for mesophyll protoplast isolation. Mature saffron leaves were divided into four segments and used to release mesophyll protoplasts (Fig. S4F). We found that the leaves in the first segment have lower moisture content, and protoplasts from this segment have a higher proportion of shrinkage (Fig. S4A). The leaves of the third and fourth segments have higher moisture content, and protoplasts from these segments have a higher proportion of rupture (Fig. S4C and D). The mesophyll protoplasts isolated from leaves of the second segment exhibited optimal characteristics, with minimal shrinkage (Fig. S4B). Thus, the leaves from the second segment were cut into 0.5–1.0 mm to optimize the best conditions for isolating mesophyll protoplasts (Fig. S4E). As mentioned above, different D-mannitol concentrations, enzyme combinations and digestion time were optimized to harvest high-yield and high-quality mesophyll protoplasts from the second segment. When the D-mannitol concentration was 0.7 M, the protoplast yield reached the peak of 7.31 × 10^5^/g FW (Fig. 4A). Eight combinations were arranged to optimize the best ratio of cellulase and macerozyme. The highest yield of mesophyll protoplasts was 6.91 × 10^5^/g FW when the enzymatic solution contained 1.5% cellulase and 0.5% macerozyme (Fig. 4C). The protoplast yield was also tested for different digestion durations, including 2 h, 3 h, 4 h and 5 h. With the extension of digestion duration, the protoplast yield increased first and then decreased. When digested for 4 h, the protoplast yield attained the highest point of 7.84 × 10^5^/g FW (Fig. 4B). Finally, the cell viability and integrity were assessed by FDA and trypan blue staining, and the results suggested that the quality of mesophyll protoplasts prepared using our method was excellent, with 96% of cells exhibiting high viability (Fig. 4E and F). The optimal conditions for mesophyll protoplast isolation involved an enzymatic solution containing 0.7 M D-mannitol, 1.5% cellulase and 0.5% macerozyme, with a digestion time of 4 h (Fig. 4A, B and C). In addition, we found that tearing the leaves into two pieces instead of cutting them into 0.5–1.0 mm segments could produce a higher yield and increased vigor of mesophyll protoplasts (Fig. S5).

### High-quality protoplasts could be used for single-cell sequencing

**Fig. 5:**
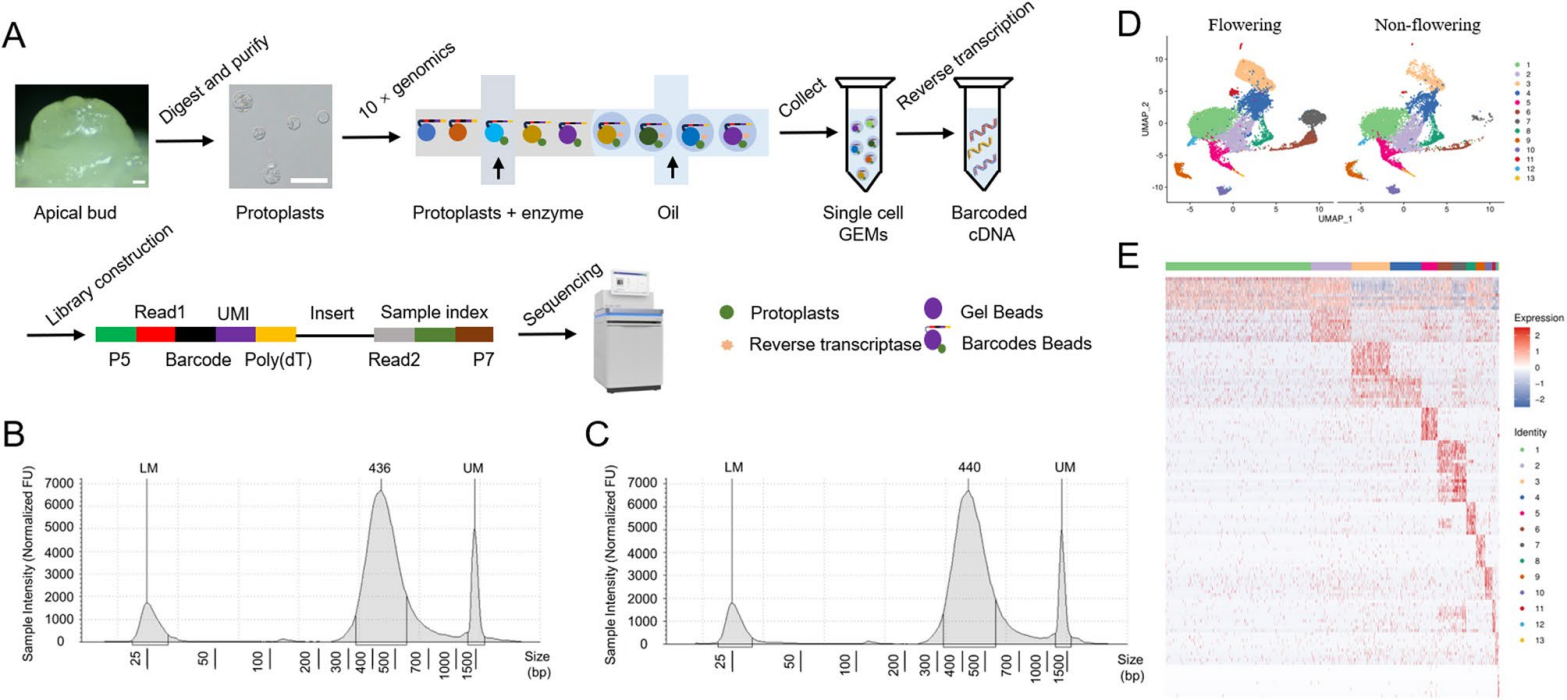
Construction of high-quality single-cell libraries. (**A**) Pipeline for constructing a scRNA-seq library using apical bud protoplasts. The fragment size distribution of scRNA-seq libraries of (**B**) Non-flowering and (**C**) Flowering apical buds was measured by the Agilent 4150 Bioanalyzer. The first and last peaks from the left in electropherograms were 25 bp (LM: Lower Marker) and 1500 bp (UM: Upper Marker) markers presented in the sample buffer, respectively. The electropherograms showed a typical smear from 300 - 600 bp, with the main peak falling around 450 bp. (**D**) Cell clustering of saffron apical bud tissue was shown. The horizontal and vertical axes represent the first and second principal components of dimensionality reduction, respectively, with distinct cell clusters distinguished by different colors. (**E**) A heatmap depicting the expression profiles of the top 10 marker genes was presented

To determine whether the protoplasts prepared by our methods could be used for single-cell sequencing, we generated two DNA-sequencing libraries at the single-cell level using protoplasts from non-flowering and flowering apical buds at the flowering transition stage (Fig. 5A). Approximately 100,000 protoplasts resuspended in 0.6 M D-mannitol were initially mixed with Master Mix, Gel Beads and partitioning oil to generate single-cell GEMs (gel bead-in-emulsion partition) (Fig. S6) by loading the mixture into Chromium microfluidic chips of a 10×Chromium Single Cell Instrument. To achieve single-cell resolution, the concentration of the cell suspension was adjusted to 1,000–1,500 cells/µL, and the volume was not less than 50 µL. The scRNA-seq library was prepared according to the manufacturer’s instructions of the Chromium Single Cell 3’ Reagent Kit v3.1. Briefly, single cells encapsulated into droplets were lysed, followed by barcoded reverse transcription of mRNA to generate the first-strand cDNA containing 10×Barcode and UMI information. The quality of scRNA-seq library was evaluated based on two metrics of fragment size distribution and the library concentration. The results showed that the fragments were distributed at 300–600 bp, with the prominent peak falling around 450 bp and the library concentration was more than 10 ng/µL (Fig. 5B and C). The libraries were sequenced by Illumina NovaSeq 6000 sequencer. 11,502 and 15,068 cells were captured to obtain a median of 2,594 and 3,869 unique molecular identifiers (UMIs) per cell for non-flowering and flowering apical buds, respectively (Fig. S7 and S8). The mean reads and median number of genes per cell were 87,735 and 1,599 for non-flowering apical buds, and 67,208 and 2,186 for flowering apical buds (Fig. S7 and S8). A total of 13 cell clusters were identified in the saffron apical bud (Fig. 5D). Among these, clusters 6, 7, and 11 were predominantly distributed in flowering apical buds, and characteristic marker genes were successfully identified for each cluster (Fig. 5D and E). In conclusion, these data indicated that protoplasts from the apical bud could be used for single-cell sequencing studies, which provided a convenient basis for deeply understanding the developmental progression of the flower primordium.

### Protein subcellular localization in saffron mesophyll protoplasts

**Fig. 6:**
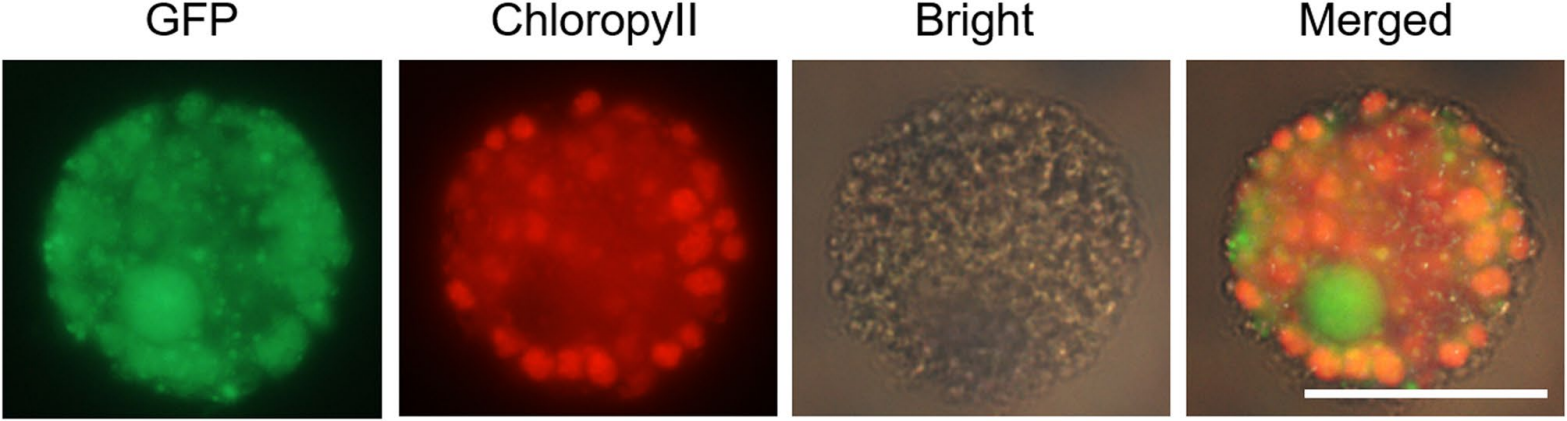
Subcellular localization analysis of GFP in protoplasts. The *p1302_35S::GFP* vector was transiently expressed in saffron mesophyll protoplasts and the signals were observed under a fluorescence microscope. Individual and merged images of GFP (green), chlorophyll autofluorescence (red) and bright field in transformed protoplasts were shown. Scale bar = 20 μm

To test whether the protoplasts prepared by our methods could be suitable for gene function identification, we performed a transient gene expression assay in mesophyll protoplasts to analyze protein subcellular localization. Heat shock combined with PEG-mediated protoplast transformation was performed using the vector *pCAMBIA1302_35S::GFP*. Strong GFP signals were distributed ubiquitously in the whole cell and noted in the nucleus, cell membrane, and cytoplasm (Fig. 6). In addition, according to the expression of GFP in the transfected protoplasts, a maximum transfection efficiency of more than 20% was obtained.

## Discussion

### Protoplast isolation from saffron: current advances and limitations

As a medicinal plant, saffron is known for its effects in promoting blood circulation and suppressing blood stasis, cooling blood detoxification, and relieving depression [36, 37]. However, due to the lack of a genetic transformation system, the functional research of target genes and germplasm resource innovation are limited in saffron. Moradi reported that saffron protoplasts prepared from style-derived calli were used to produce crocin and phenolics [38], but preparing callus tissue was laborious and time-consuming. In our study, we reported the methods of saffron apical bud, root, petal, and mesophyll protoplast isolation. The protoplast yield from petals (~ 1.36 × 10⁷/g FW) in our study is comparable to that reported for rice leaves (~ 1.0 × 10⁷/g FW) [16] and maize leaves (~ 1.8–1.9 × 10⁷/g FW) [39], yet lower than that of Chinese kale mesophyll protoplasts (6.04 × 10⁷/g FW) [40]. In contrast, yields from saffron apical buds (~ 8.81 × 10^6^/g FW) and mesophyll tissue (~ 7.84 × 10⁵/g FW) were lower than petal yields, reflecting the inherent challenges of isolating protoplasts from mature, differentiated monocot tissues enriched in sclerenchyma fibers and secondary cell wall thickenings, a limitation similarly encountered in orchid protoplast isolation (~ 3.22 × 10⁶/g FW) [41]. Furthermore, our findings corroborate previous observations that optimal enzymatic digestion conditions are highly species- and tissue-specific.

Nonetheless, several limitations persist, including the scalability of these methods for high-throughput applications, the long-term maintenance of protoplast cultures, and the efficiency of plant regeneration from protoplasts, which have yet to be fully optimized. Future investigations should systematically address these constraints through multi-dimensional optimization of culture media formulations and environmental parameters, development of novel strategies to sustain mitotic activity over extended culture periods, and establishment of universally applicable, genotype-independent regeneration protocols. Overcoming these technical hurdles will substantially expand the utility of protoplast-based approaches in plant biotechnology and functional genomics, thereby facilitating advancements in genetic engineering and germplasm innovation.

### Effects of developmental stage and sampling location on protoplast yield and quality

The developmental stage, sampling location and sample handling used in protoplast isolation can greatly influence the yield and quality of the protoplasts [42]. For example, protoplasts isolated from the apical buds at the later stage of flower organ differentiation were observed to have a greater abundance of debris compared to apical buds at the flowering transition stage. The protoplasts isolated from the white petals were easy to rupture, while the protoplasts isolated from the purple petals had a higher pigment content, which affected the implementation of subsequent molecular biology experiments. Mature saffron leaves were divided into four segments, and only the second segment was used to isolate the highest yield of protoplasts with the best cell viability. In our study, we found that tearing the leaves into two pieces instead of cutting them into 0.5–1 mm segments could produce a higher yield and increased vigor of mesophyll protoplasts (Fig. S5). Furthermore, the yield and quality of isolated protoplasts varied across tissue types. Apical buds, petals, and leaves yielded high-quality protoplasts with approximately 95% viability, whereas root-derived protoplasts exhibited markedly reduced viability of only 61%. Compared with leaves and roots, apical buds and petals produced higher protoplast yields, reaching 8.81 × 10^6^ and 1.36 × 10⁷/g FW, respectively. Taken together, the yield and quality of protoplasts derived from apical buds, petals, and leaves are considered satisfactory for subsequent molecular biology experiments.

The pronounced variation in protoplast yield and quality, depending on developmental stage and sampling location, likely arises from intrinsic tissue-specific characteristics in plants. Cell wall composition undergoes substantial changes during development and varies considerably among organs [43, 44], leading to differential accessibility of cell wall components to hydrolytic enzymes. For example, the high viability of protoplasts derived from apical buds, young leaves, and petals may be attributed to their predominantly primary cell walls, which are rich in pectin and cellulase but low in lignin, thereby facilitating efficient enzymatic digestion. In contrast, the lower yields and viability observed in root tissues could result from more recalcitrant cell wall components or a higher proportion of cells undergoing secondary wall thickening. Furthermore, variations in basal metabolic activity among tissues [45] may precondition cells to better withstand the rigors of the isolation process. Future studies should explore how tissue-specific differences in cell wall composition and metabolic status influence protoplast yield and quality, thereby providing essential theoretical guidance for establishing robust and efficient protoplast isolation protocols across diverse species and tissues.

### Technical challenges and optimization strategies for protoplast-based scRNA-seq in plants

Single-cell RNA sequencing technology is a powerful tool for studying plant development trajectories and environmental cues. However, preparing high-quality protoplasts is a prerequisite for single-cell sequencing. Firstly, due to the uneven thickness and different components of the cell wall in different species and tissues, the D-mannitol concentration, enzyme combinations and digestion time need to be explored by researchers [41, 42], which greatly increases the difficulty of the experiment. Secondly, the enzymatic removal of cell walls may potentially perturb cellular transcriptional programs to varying extents, which could consequently introduce transcriptional artifacts that complicate scRNA-seq data interpretation. Our protoplast isolation protocol attempts to address this concern by restricting enzymatic digestion to less than 4 h, yielding protoplasts with ~ 95% viability that appear to retain the majority of their biochemical and cellular functions. Importantly, parallel profiling of stress-responsive genes in intact, non-digested tissues may allow direct comparison with scRNA-seq datasets, potentially enabling quantitative assessment of protoplast stress levels and more accurate inference of gene expression patterns [46]. Thirdly, cell size and viability also significantly impact the quality of single-cell sequencing. Unlike animal cells, plant cells can be up to 100 μm in diameter. The cell size not only affects cell clustering and cell type classification but also affects the identification of differentially expressed genes (DEGs). The 10×Genomics single-cell transcriptome sequencing platform specifies that the cell size cannot exceed 50 μm to reduce size-biased quantification of transcriptional activity [47]. Furthermore, Ca^2+^ and Mg^2+^ contained in the solution can interfere with the reverse transcription reaction, so protoplasts used to construct single-cell libraries should be resuspended in D-mannitol solution [42]. Appropriate D-mannitol concentration can maintain the interior and exterior osmotic pressure of the cells, preventing them from rupturing or collapsing.

### Comparison between scRNA-seq and snRNA-seq

As emerging technologies, single-nucleus RNA sequencing (snRNA-seq) and scRNA-seq are widely used in the study of developmental biology both in animals and plants. Each method possesses distinct advantages and limitations, necessitating researchers to select the appropriate technology that aligns with their specific requirements and circumstances. When performing scRNA-seq, enzymatic digestion is required to lyse fresh material to obtain single-cell suspensions, potentially leading to changes in the expression of stress genes and affecting the reliability of the data [48, 49]. In contrast, snRNA-seq can use fresh, fixed and frozen samples to prepare single-cell nuclear suspensions by isolating cell nuclei, which avoids the bias of data due to the stress reaction caused by the enzymatic digestion method [50, 51]. Protoplasts are prone to generating cellular debris during preparation, and they contain secondary metabolites that may affect the reverse transcription and amplification processes [52, 53]. For scRNA-seq, cells exceeding 40 μm in size cannot pass through the channels within 10×Genomics microfluidic chip. However, for snRNA-seq, there is no need to take cell size into account. The primary advantage of scRNA-seq over snRNA-seq is that it captures information from the entire cell, whereas snRNA-seq can only capture information in the nucleus and lacks information in the cytoplasm [50]. Nevertheless, it has been reported that the gene detection sensitivity of single-nucleus platforms is on par with that of single-cell platforms [54]. In general, scRNA-seq is preferred if high-quality single-cell suspensions can be prepared. In this study, we prepared high-quality saffron protoplasts derived from different organs, with only 3% of cells exceeding 40 μm in size, which can be given priority for single-cell transcriptomic analysis in exploring the developmental biology and molecular mechanisms of saffron.

### Establishment of an efficient protoplast transient expression system in *Crocus sativus* L.

The protoplast-based transient expression system is a very useful tool for gene function identification, such as protein subcellular localization, protein-protein interactions and gene regulation. This system avoids the interference caused by a heterologous system. The PEG-mediated transformation has been successfully applied to protoplast transformation in a variety of plants [7, 29, 41, 42]. However, no matter how the PEG concentration, plasmid concentration and transformation time were adjusted, successfully transformed protoplasts could not be obtained in saffron. In our study, we performed the protoplast transformation using heat shock combined with PEG-mediated method. We transfected saffron mesophyll protoplasts with a GFP-expressing plasmid to verify that the transient expression system was functional. The transformation efficiency in saffron mesophyll protoplasts reached up to 20%, which may only satisfy the protein subcellular localization assay. However, efforts will be made to further improve the transformation efficiency in future work.

Considering the limited research advancements in saffron’s developmental biology, the proposed tissue-specific protoplast isolation technique holds promise for cultivating novel germplasm with superior traits and facilitating the identification of gene functions and signaling pathways in saffron.

In this study, scRNA-seq and transient expression assay were performed using high-quality protoplasts, which will help botanists accelerate research on germplasm resource innovation and molecular mechanisms involved in regulating organogenesis, growth and development regulation of saffron.

## Conclusions

The yield, quality, and optimal isolation conditions of protoplasts varied across tissue sources. Protoplasts derived from apical buds, petals, and leaves exhibited high viability (> 90%), whereas those from roots showed substantially lower viability (~ 60%). For petals and leaves, the developmental stage and sampling position were critical factors influencing protoplast yield and quality. Specifically, the light-purple petal and the second segment of leaves produced protoplasts with higher yield and better quality. Building upon these optimized conditions, we constructed two high-quality single-cell transcriptomic libraries from non-flowering and flowering apical buds, respectively. Specific cell clusters and marker genes were successfully identified in the flowering apical buds. Furthermore, the subcellular localization of GFP in mesophyll protoplasts was also successfully visualized. Collectively, the establishment of tissue-specific protoplast isolation methods will accelerate both germplasm innovation and mechanistic understanding of organogenesis, growth, and developmental regulation in saffron.

## Supplementary Information

Below is the link to the electronic supplementary material.

Supplementary Material 1

Supplementary Material 2

## Data Availability

Supplementary data generated or analyzed during this study are included in this article. The raw reads generated here have been submitted to the NCBI BioProject database (https://www.ncbi.nlm.nih.gov/bioproject/) under accession numbers PRJNA1457925 and PRJNA1457928.
