# Supplementary material for: An efficient method for tissue-specific protoplast isolation suitable for single-cell RNA sequencing and transient gene expression analysis in saffron (Crocus sativus L.)
Source: BMC Biotechnol. 2026 Jul 8;26:87. doi: 10.1186/s12896-026-01187-1 (PMC13352814; doi:10.1186/s12896-026-01187-1)
Supplement: Supplementary file 1 — Supplementary Material 1 [file 12896_2026_1187_MOESM1_ESM.docx]

**Supplementary Information**


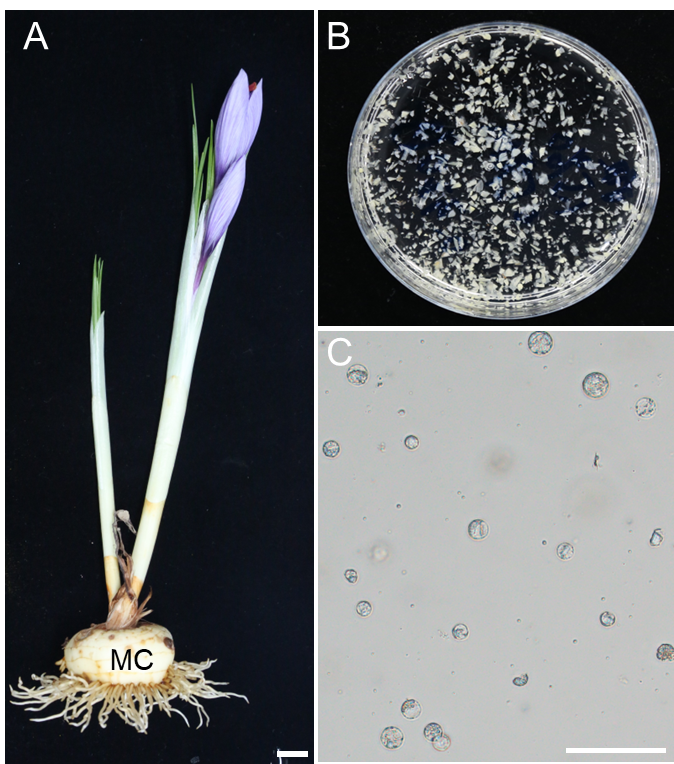


**Fig. S1 Establishment of a method for apical bud protoplast isolation.** (A) A saffron plant with flowering competency was shown, and its propagation was through mother corms. MC: mother corm. Scale bar = 1 cm. (B) Cut apical buds into 0.5-1.0 mm strips using fresh surgical blades in the petri dish and immerse them in the enzymatic solution. (C) The protoplasts isolated from apical buds under brightfield were shown. Scale bar = 100 µm.


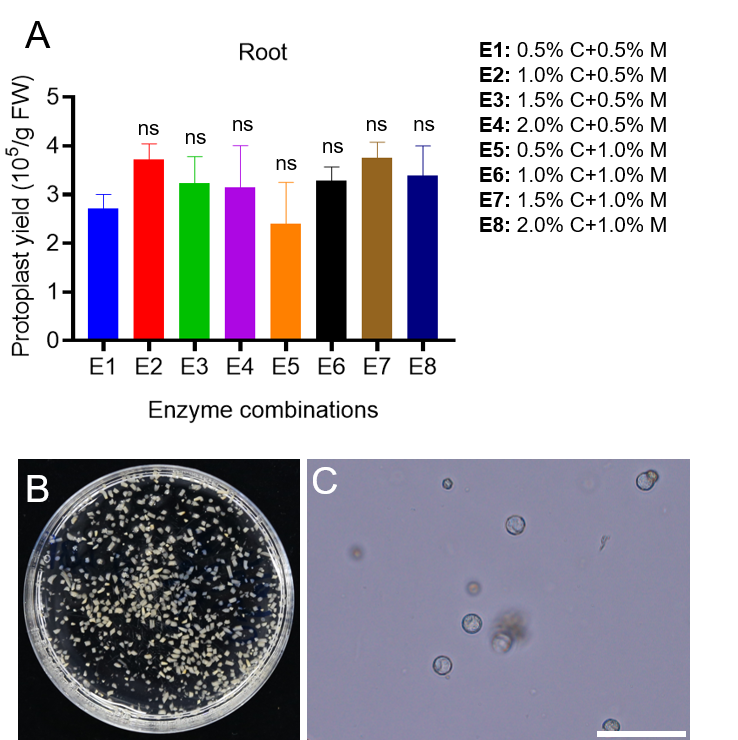


**Fig. S2** **Establishment of a method for fibrous root protoplast isolation.** (A) Effects of different enzyme combinations on the yield were shown. C: cellulase R-10, M: macerozyme R-10. Data are means ± SD of three biological replicates. *P < 0.05, **P < 0.01, ***P < 0.001; ns, not significant. (B) Cut fibrous root tips into 0.5-1.0 mm strips using fresh surgical blades in the petri dish and immerse them in the enzymatic solution. (C) The protoplasts isolated from fibrous roots under brightfield were shown. Scale bar = 100 µm.


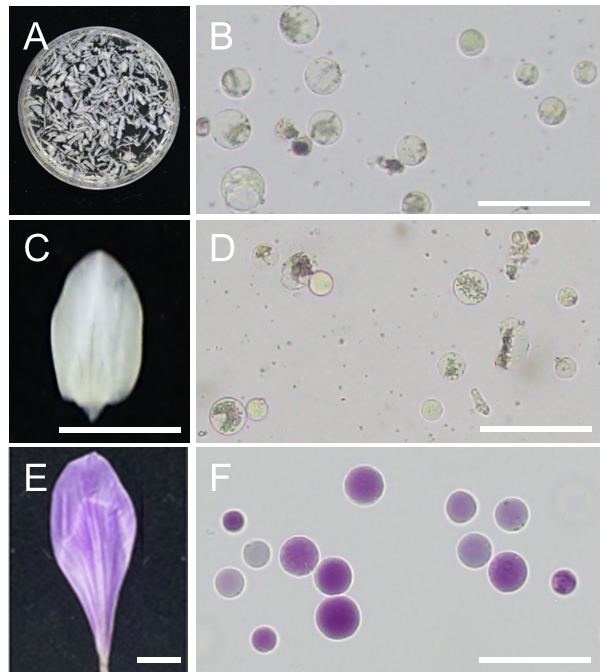


**Fig. S3 Establishment of a method for petal protoplast isolation.** (A) Cut light purple petals into 0.5-1.0 mm strips using fresh surgical blades in the petri dish and immerse them in the enzymatic solution. (B) The protoplasts isolated from light purple petals under brightfield were shown. (C) White petal and (D) the protoplasts released from them under brightfield were shown. (E) Dark purple petals and (F) the protoplasts released from them under brightfield are shown. Scale bar = 100 µm for (B), (D) and (F). Scale bar = 0.5 cm for (C) and 1 cm for (E).

**
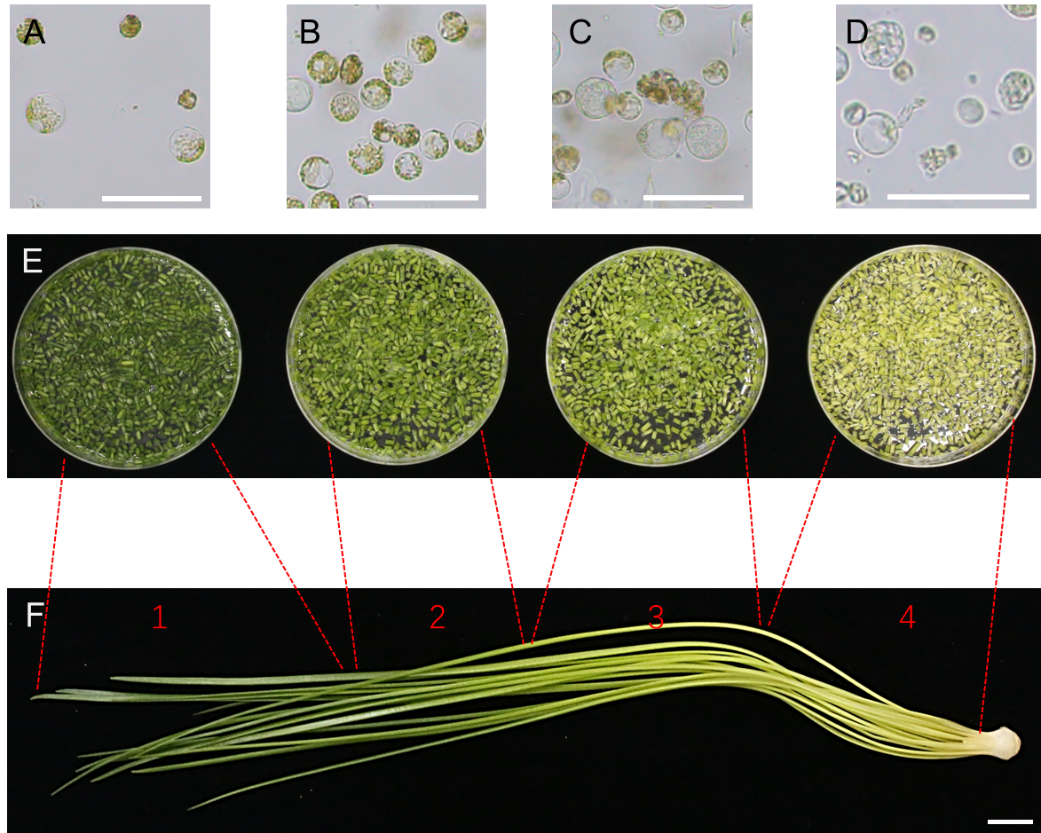
**

**Fig. S4 Establishment of a method for** **mesophyll protoplast isolation.** (A, B, C, D) The quality of mesophyll protoplasts released from segments one, two, three and four of saffron leaves was shown. Scale bar = 100 µm. (E) Cut segments 1-4 into 0.5-1.0 mm strips using fresh surgical blades in the petri dish and immerse them in the enzymatic solution. (F) The leaves of saffron were divided into four segments for mesophyll protoplast preparation. Scale bar = 1 cm.


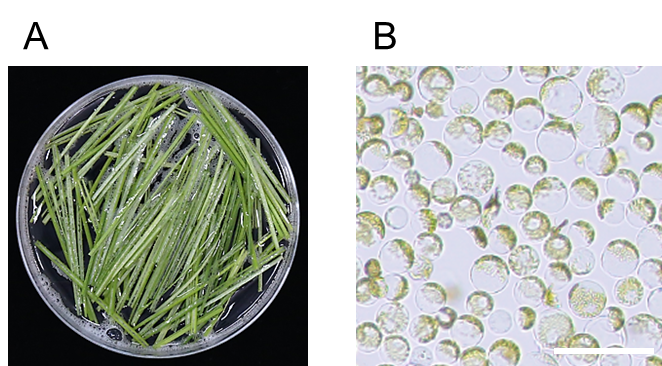


**Fig. S5** **High-yield and -quality protoplasts were harvested when tearing the leaves into two pieces.** (A) The leaves were first torn into two pieces, then further cut into 4-5 cm segments, and finally immersed in an enzymatic solution. (B) The mesophyll protoplasts harvested from (A) were shown. Scale bar = 100 µm.


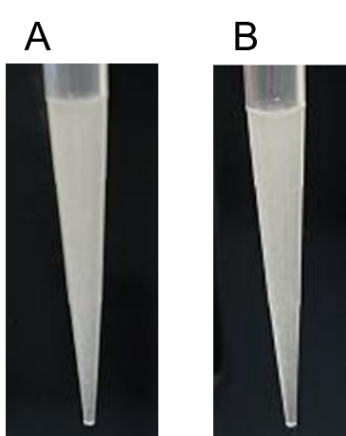


**Fig. S6 The generation of single-cell GEMs (Gel Bead-In-Emulsions) for protoplasts isolated from (A) the non-flowering and (B) flowering apical buds at the flowering transition stage.**

**
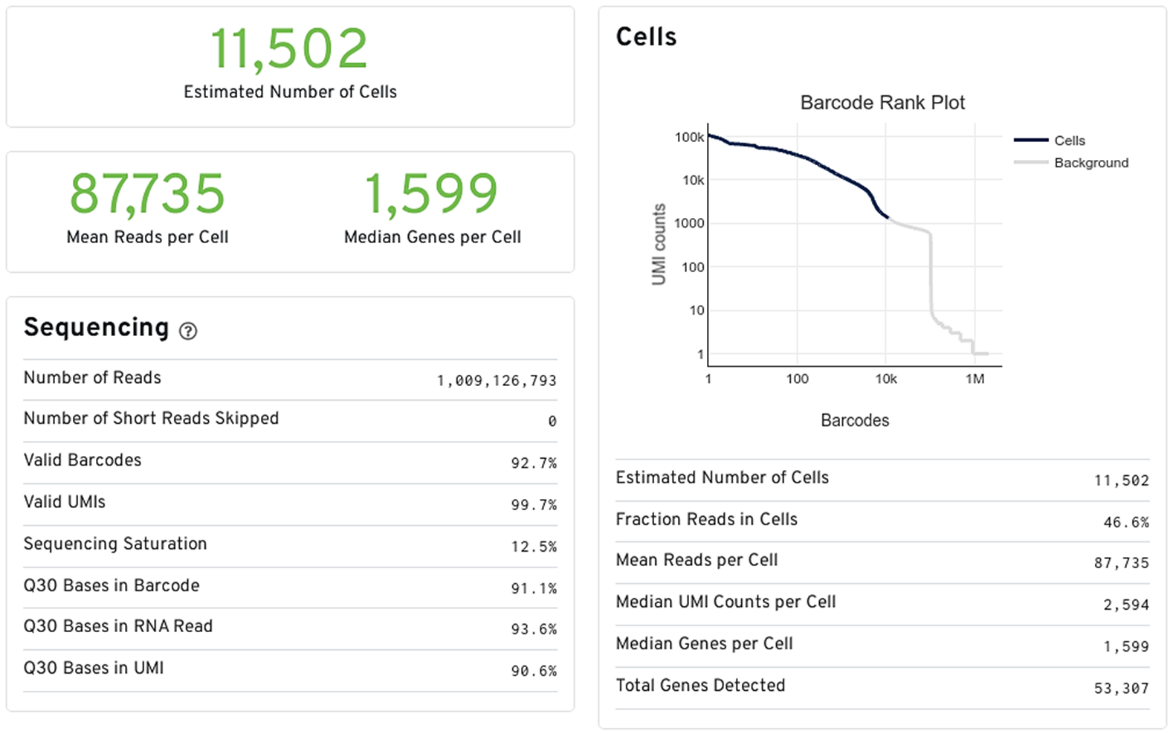
**

**Fig. S7 The quality control results of the non-flowering apical bud library were shown.** The library was sequenced by Illumina NovaSeq 6000 sequencer with 2 × 150 bp paired-end reads with a unique dual index.

**Fig. S8 The quality control results of the flowering apical bud library were shown.** The library was sequenced by Illumina NovaSeq 6000 sequencer with 2 × 150 bp paired-end reads with a unique dual index.**
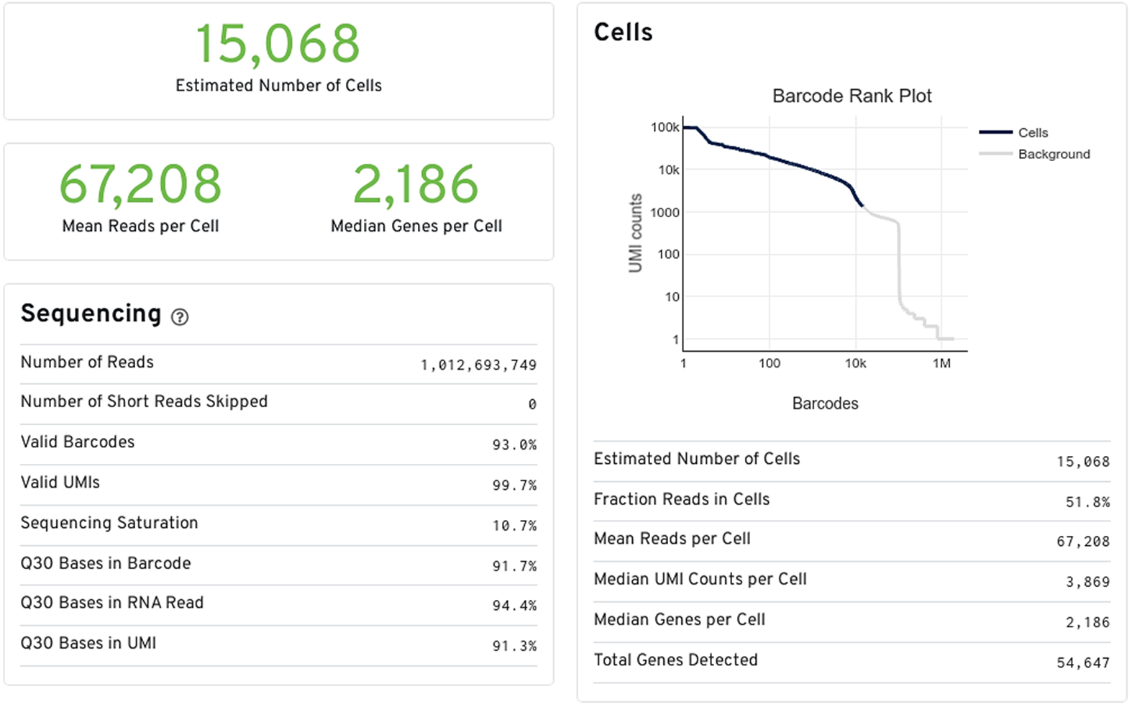
**
